# Palmitate and Stearate are Increased in the Plasma in a 6-OHDA Model of Parkinson’s Disease

**DOI:** 10.3390/metabo9020031

**Published:** 2019-02-13

**Authors:** Anuri Shah, Pei Han, Mung-Yee Wong, Raymond Chuen-Chung Chang, Cristina Legido-Quigley

**Affiliations:** 1 Institute of Pharmaceutical Science, Faculty of Life Sciences and Medicine, King’s College London, London SE19NH, UK; anuri.shah@hku.hk (A.S.); hanpei0126@hotmail.com (P.H.); 2 Laboratory of Neurodegenerative Diseases, School of Biomedical Sciences, LKS Faculty of Medicine, The University of Hong Kong, Hong Kong, China; mungyee.wong@gmail.com; 3 Institute of Materia Medica, Chinese Academy of Medical Sciences & Peking Union Medical College, Beijing 100006, China; 4 State Key Laboratory of Brain and Cognitive Sciences, The University of Hong Kong, Hong Kong, China; 5 Steno Diabetes Center Copenhagen, DK-2820 Gentofte, Denmark

**Keywords:** Parkinson’s disease, 6-OHDA, GC-MS, plasma, midbrain, fatty acid metabolism, myo-inositol

## Abstract

Introduction: Parkinson’s disease (PD) is the second most common neurodegenerative disorder, without any widely available curative therapy. Metabolomics is a powerful tool which can be used to identify unexpected pathway-related disease progression and pathophysiological mechanisms. In this study, metabolomics in brain, plasma and liver was investigated in an experimental PD model, to discover small molecules that are associated with dopaminergic cell loss. Methods: Sprague Dawley (SD) rats were injected unilaterally with 6-hydroxydopamine (6-OHDA) or saline for the vehicle control group into the medial forebrain bundle (MFB) to induce loss of dopaminergic neurons in the substantia nigra pars compacta. Plasma, midbrain and liver samples were collected for metabolic profiling. Multivariate and univariate analyses revealed metabolites that were altered in the PD group. Results: In plasma, palmitic acid (*q* = 3.72 × 10^−2^, FC = 1.81) and stearic acid (*q* = 3.84 × 10^−2^, FC = 2.15), were found to be increased in the PD group. Palmitic acid (*q* = 3.5 × 10^−2^) and stearic acid (*q* = 2.7 × 10^−2^) correlated with test scores indicative of motor dysfunction. Monopalmitin (*q* = 4.8 × 10^−2^, FC = −11.7), monostearin (*q* = 3.72 × 10^−2^, FC = −15.1) and myo-inositol (*q* = 3.81 × 10^−2^, FC = −3.32), were reduced in the midbrain. The liver did not have altered levels of these molecules. Conclusion: Our results show that saturated free fatty acids, their monoglycerides and myo-inositol metabolism in the midbrain and enteric circulation are associated with 6-OHDA-induced PD pathology.

## 1. Introduction

Parkinson’s disease (PD) affects approximately 1% of the population above the age of 50 years, worldwide [1]. Costs of treatment per capita in the U.K. alone can be up to £13,804 annually [2]. Aging populations are generally at greatest risk [3]. Up to 18 genetic loci have been demonstrated to contribute to familial cases of the disease [4]. It is estimated that the incidence of PD worldwide will double within the next decade [1]. All these factors combined are increasingly directing research and development not only towards novel therapies, but also technologies for better diagnosis and disease management. Diagnosis of PD is primarily based on clinical symptoms. However, the error rate is high [5]. Of these, the main features used for clinical diagnosis are heavily relied on symptoms such as bradykinesia, rigidity, tremors, postural instability and freezing [6].

Metabolomics is a well-defined approach used for biomarker discovery and investigating disease mechanisms. Burté et al. undertook metabolomic profiling of the serum samples of early stage PD patients and found an increase in metabolites of the fatty acid beta oxidation pathways [7]. In one longitudinal study, it was found that a combination of plasma and CSF xanthine and fatty acid metabolites showed significant changes between baseline and the study end point (two years from baseline) [8]. Markers that were highly correlated with a change in UPDRS scores, indicative of PD progression, included benzoate in the CSF and phenylcarnitine and aspartylphenylalanine in the plasma. Several studies on urine samples of PD patients showed an increase in amino acid metabolism, including phenylalanine [9], histidine, glycine and tryptophan/kynurenine [10]. A study by Ohman et al., using NMR based metabolomics on CSF, demonstrated the role of the amino acid alanine, energy metabolism (creatinine) and glucose metabolism (mannose) in distinguishing PD patients from controls [11]. Metabolic profiling of CSF has also been useful in differentiating newly diagnosed PD patients from controls. Trupp et al. revealed an increase in levels of the amino acids, alanine and methionine, and a reduction of saturated and unsaturated fatty acids in the CSF of newly diagnosed PD patients [12]. A differential role of glutathione metabolism has also shown a link to PD. While one study found increased glutathione in the plasma [5], another study revealed lower oxidized glutathione levels in the CSF [13], further suggesting the involvement of free radicals in PD.

A wide range of in vivo transgenic- and toxin-based models of PD are available. Studies on the mesencephalon of MPTP-induced PD in mice have revealed the role of altered energy [14], ceramide and sphingolipid metabolism [15]. A range of lipid species, including lysophosphatidylcholines and phosphatidylcholines are shown to be involved in 6-OHDA toxicity in rat midbrains [16]. An MPTP-treated goldfish model also indicated the role of phosphocholine metabolism, along with amino acids such as leucine, valine and glutamine [17]. Additionally, cardiolipins and mitochondria-associated phospholipids were detected in mesencephalon and plasma samples of rotenone-treated rats [18].

The aim of this study is to discover metabolic pathways in different tissues in a unilateral PD model. Plasma and liver profiling are often used as an indicator of metabolic changes, whereas in this case the midbrain is the site of primary pathological changes in the brain.

## 2. Results

### 2.1. Validation of the 6-OHDA Model

**Figure 1:**
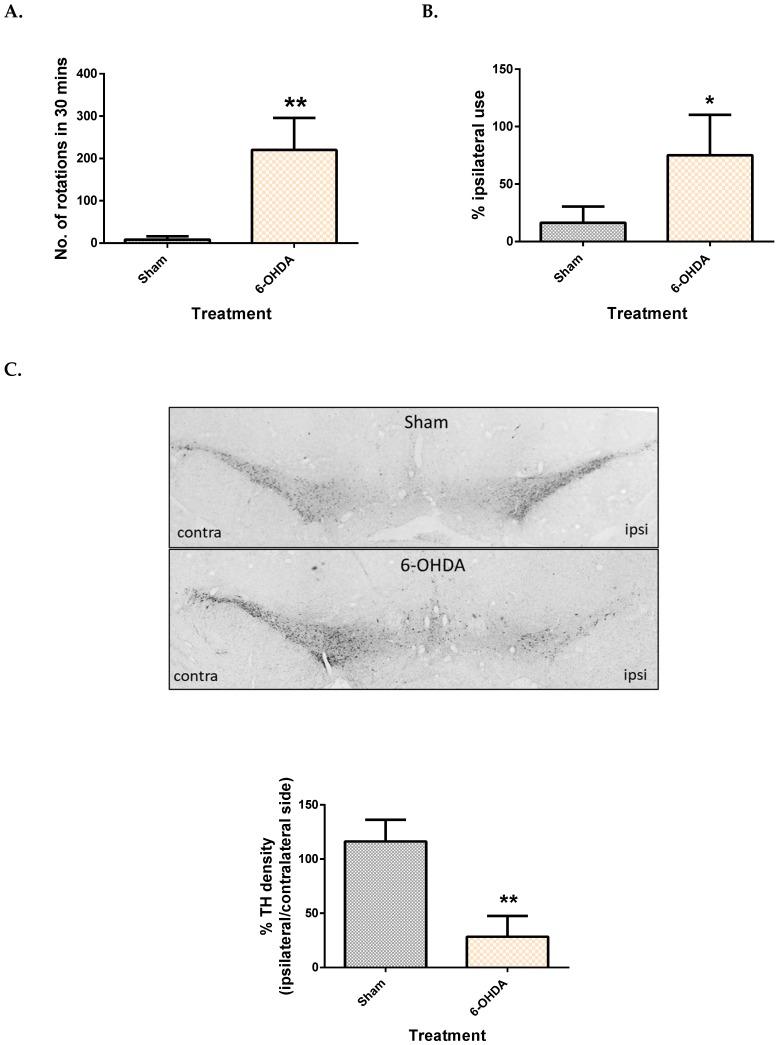
Comparison of motor function and dopaminergic cell loss between control and 6-OHDA groups. Apomorphine induced rotation test (**A**), Cylinder test (**B**) and TH density (**C**) between the sham and 6-OHDA group. Contra = contralateral and ipsi = ipsilateral. Data represent mean ± S.D of at least 5 rats in each group. (* indicates *p* value < 0.05 and ** *p* < 0.01, using Mann–Whitney test.).

The apomorphine-induced rotation test was used to monitor the intensity of the ipsilateral lesion, while the cylinder test was used to assess motor dysfunction presented by asymmetry in the forepaw use of the rats. It was observed that the number of contralateral rotations in 30 min was significantly higher in rats that received 6-OHDA, with an average of 220 rotations (Figure 1A). Rats that received 6-OHDA injection showed an inability to use the contralateral limb while rearing, compared to their sham counterpart. This was evidenced by a 75% dependence on the ipsilateral limb while rearing (Figure 1B).

After behavioural testing, immunohistochemistry was done to confirm loss of dopaminergic neurons in the SNpc by counting TH immuno-positive neurons. It was observed that rats in the sham group had a similar TH count on both sides (Figure 1C), while the 6-OHDA group showed only 28% TH density on the ipsilateral side compared to the contralateral side (Figure 1C).

### 2.2. Metabolomic Method Validation and Feature Selection

The metabolomics workflow is described in Supplementary Figure S2. To assess the reproducibility of our analysis, PCA plots were used. QC samples in both the plasma and the midbrain plots clustered together showing good repeatability (supplementary Figure S3). Up to 1500 metabolic features in the plasma and 2500 metabolic features in the mesencephalon regions were obtained.

OPLS-DA multivariate analysis showed a significant separation between the sham and 6-OHDA groups in the plasma and midbrain (supplementary Figure S4). Corresponding S-plots then revealed 16 metabolic features (4 from plasma tissues, and 12 from midbrain) altered between the 6-OHDA and sham groups. After comparing to NIST library, 13 were identified with similarity index >85%, two were identified as sugars and one remained unknown.

### 2.3. Metabolite Levels in the Plasma, Brain and Liver

**Figure 2:**
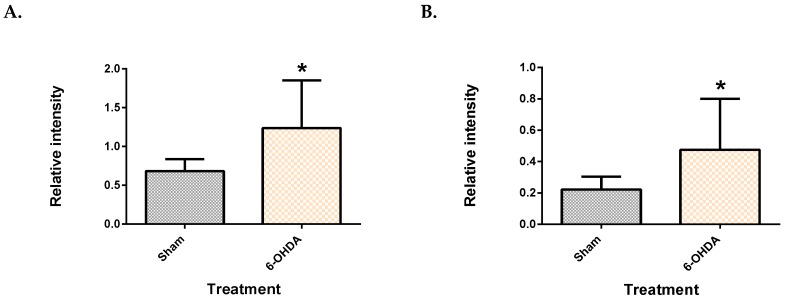
Saturated free fatty acids in the plasma. Palmitic acid (**A**) and stearic acid (**B**) were upregulated in the plasma of 6-OHDA-lesioned rats. Data represent mean ± S.D of at least 5 animals in each group. (* indicates *q* value < 0.05 using Mann–Whitney test, followed by Benjamini–Hochberg correction.).

Five features (two from the plasma, and three from the brain) showed significant difference between the groups after Benjamini–Hochberg correction. The two plasma metabolite features, which were identified as palmitic acid and stearic acid (similarity index >90%), were significantly upregulated in 6-OHDA group (Figure 2), compared to the sham (*q* = 3.72 × 10^−2^ for palmitate and *q* = 3.84 × 10^−2^ for stearate). Post-hoc power analysis yielded a statistical power of 93.2% for palmitic acid and 86.5% for stearic acid.

**Figure 3:**
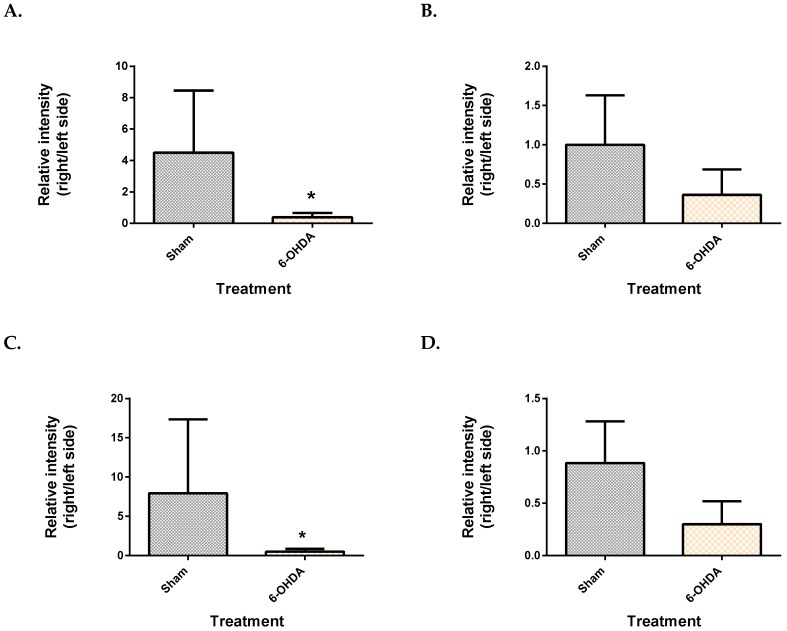

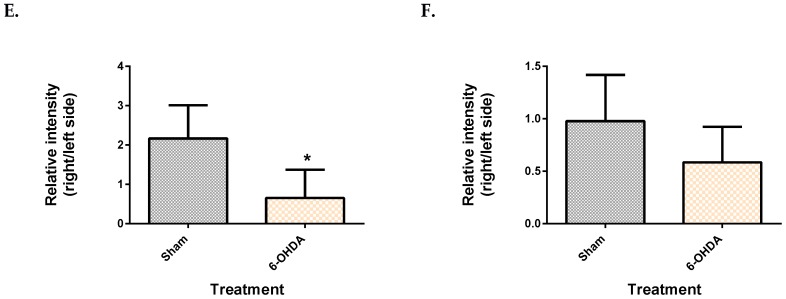
Comparison of brain metabolite changes between the mesencephalon and cerebellum. Midbrain monopalmitin (**A**), monostearin (**C**) myo-inositol (**E**) were significantly altered while cerebellar monopalmitin (**B**), monostearin (**D**), myo-inositol (**F**) and) were unchanged. Data represent mean ± S.D of at least 5 animals in each group. (* indicates *q* value < 0.05 using Mann–Whitney test, followed by Benjamini–Hochberg correction).

From the mesencephalon, all three metabolite features presented lower levels in the 6-OHDA group compared to the sham (Figure 3). These were identified as monopalmitin (*q* = 4.8 × 10^−2^), monostearin (*q* = 3.72 × 10^−2^) and myo-inositol (*q* = 3.81 × 10^−2^). Monopalmitin and monostearin had a similarity index of more than 90%, while myo-inositol was 88%. The myo-inositol pure standard confirmed the identity of myo-inositol (supplementary Figure S5). Post-hoc power analysis revealed that the two monoglycerides showed a statistical power below 80% while myo-inositol had a statistical power of 97.4%.

**Figure 4:**
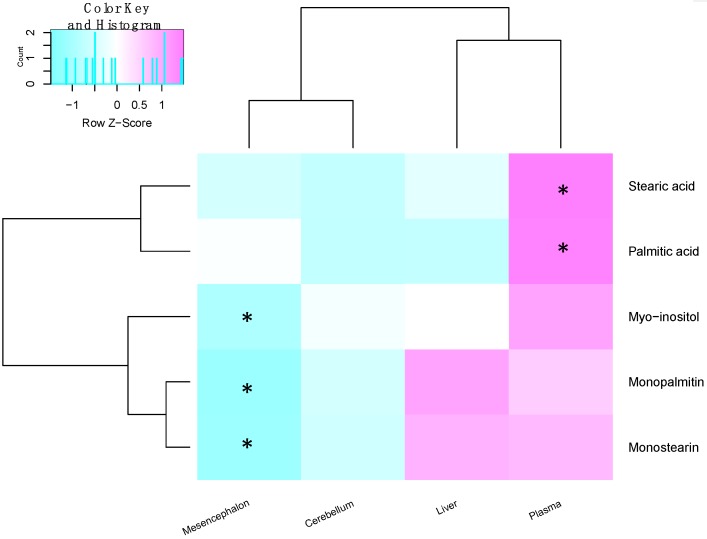
Heat map showing the fold change of metabolites between the sham and 6-OHDA groups within the different tissues. Similarly changed metabolites are clustered together, while tissues with similar changes in metabolites are near to each other. Significantly changed metabolites between the sham and 6-OHDA treated rats are marked for each tissue. (* indicates *p* < 0.05 after Benjamini–Hochberg correction, in that tissue.

**Figure 5:**
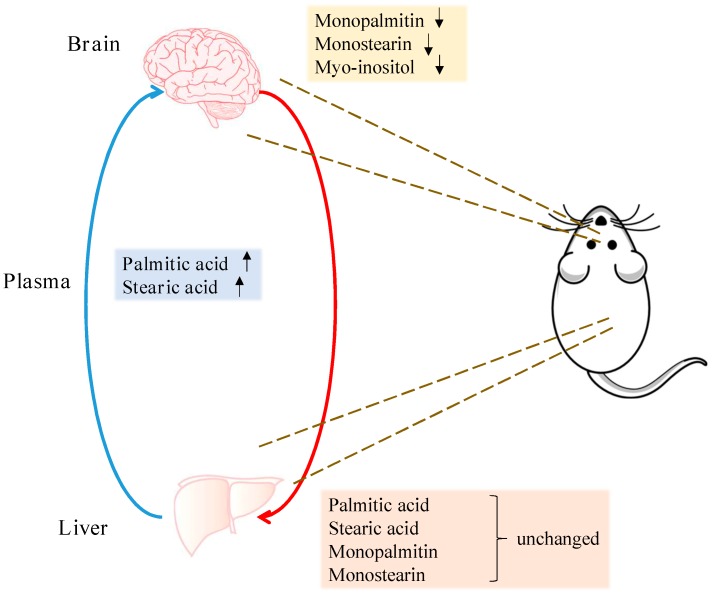
Summary of region-specific metabolite changes. Arrows indicate metabolites significantly increased or decreased in the midbrain and plasma of the 6-OHDA-treated animals. Levels of these metabolites remained unchanged in the liver.

The same univariate approach was applied to liver palmitic acid, stearic acid, monopalmitin and monostearin levels. The levels of these four metabolites remained unchanged in the liver, between the 6-OHDA and the sham groups (Supplementary Figure S6). A summary of all metabolite changes has been illustrated in Figure 4 and Figure 5.

### 2.4. Correlation of Plasma and Midbrain Features with Motor Dysfunction

Spearman’s correlation was done to gauge the relationship between the levels of all five features and the motor impairment examined by the behaviour tests Table 1. The plasma metabolites were found to be highly correlated with motor dysfunction. Palmitic acid showed a strong positive correlation (r = 0.674, *q* = 0.035) with rigidity in the contralateral forelimb movement, as examined by the cylinder test. Stearic acid also had a high positive correlation with the cylinder test (r = 0.649, *p* = 0.027).

## 3. Discussion

The aim of this study was to elucidate metabolite changes in an in vivo model of PD. To confirm the effectiveness of our model, we performed behaviour tests and immunohistochemistry to examine the loss of dopaminergic neurons in the SNpc. Unilateral lesions of 6-OHDA successfully resulted in the manifestation of motor symptoms, as observed by the cylinder test, and the apomorphine-induced rotation test indicating the intensity of the lesions. Additionally, a significant loss of dopaminergic neurons was observed on the ipsilateral side, as measured by counting TH immuno-reactive positive cells. All these results proved the validity of a “hemi-parkinsonian” model.

Palmitic acid and stearic acid were significantly increased in the plasma, while an imbalance of their monoglyceride forms in the midbrain was also observed. Fatty acids can be transported through the blood–brain barrier via two major routes, either passive diffusion [19], or facilitated by transporters [20]. The transport of these two upregulated fatty acids in the context of PD needs to be further studied. One study has shown a decrease of palmitic and linoleic acid in the human plasma [12]. Moreover, the significant correlation between plasma palmitate and stearate and the cylinder test is of particular interest, suggesting an association with symptoms. This association of the fatty acids with motor symptoms is indicative of the severity of the 6-OHDA lesion and thus damage induced by it. Owing to a lack of biomarkers for PD, diagnosis currently relies heavily on symptoms. It is worthwhile to investigate the clinical potential of these fatty acids in PD, further. Moreover, longitudinal studies will aid in assessing whether palmitate and stearate levels change as neuronal damage progresses in this model. Finding a biomarker that correlates with worsening of symptoms is ideal for tracking disease progression.

Saturated free fatty acids are released into the blood by two major pathways. Lipolysis [21] is the breakdown of fats in adipose tissue to release triglycerides and free fatty acids into the blood, whereas de novo lipogenesis (DNL) occurs when saturated fatty acids are synthesized from glucose and its metabolites in the liver are subsequently released into the plasma to target tissues in need. There is also evidence to show that palmitic acid may be the major product of DNL [22]. However, our results show that there was no increase of palmitic or stearic acid in the liver tissue. This observation can be attributed to a swift clearance of the liver fatty acids by the plasma. In addition, oxidation of fatty acids, which takes place in the mitochondria, is an important pathway providing energy [23]. Mitochondrial dysfunction has also been implicated in PD [24]. The increased levels of stearic acid and palmitic acid in plasma could, therefore, be a consequence of impaired mitochondria in hepatic or extrahepatic tissues.

Saturated free fatty acids have known effects in the context of neuronal conditions. In one study, a diet rich in palmitic acid (30% palmitic acid of total fat) fed to mice resulted in reduced hippocampal neurogenesis [25]. Additionally, a diet supplemented with palmitate (2.2% *w*/*w*) induced endoplasmic reticulum (ER) stress in murine hippocampi and cortices. This study also assessed effects of increasing concentrations of palmitate, of up to 500 µM, on human neuroblastoma SH-SY5Y cells, which are commonly used for PD studies. It was found that 100 µM and higher concentrations of palmitic acid led to an upregulation of the ER stress-associated pro-apoptotic signalling machinery CHOP [26]. Additionally, 0.2 mM palmitic and stearic acid induced hyperphosphorylation of tau in rat primary cortical neurons, which was facilitated by astrocyte-induced oxidative stress [27]. Hyperphosphorylation of tau is a hallmark of Alzheimer’s disease. An increased uptake of labelled palmitate into the brain was also observed in patients with metabolic syndrome compared to that of control subjects. Elevated levels of free fatty acids in the plasma have also been linked to metabolic syndrome [28,29,30,31,32]. This is particularly important given that PD affects an aging population, many of which suffer from metabolic syndrome as well.

On the other hand, monoglycerides of palmitic acid and stearic acid had a significant difference between the lesioned and non-lesioned sides of the mesencephalon, with no corresponding changes in the cerebellum. The cerebellum was used as a control region because this region remains unaffected by 6-OHDA lesions into the MFB. Monoglycerides are an intermediate product formed during the breakdown of triglycerides by lipolysis [33]. Whether the imbalance of these metabolic features can be stem from the imbalance of dopaminergic neurons must be further assessed. Additionally, the corresponding levels of these metabolites in the striatal terminals will give a more holistic idea about their association with dopaminergic loss; 1-monopalmitin and 1-monostearin have been shown to be altered in the CSF of patients with inflammatory demyelinating disease such as multiple sclerosis, a disorder affecting nerve fibres [34].

In this study, myo-inositol also showed an imbalance in the mesencephalon. Studies on the basal ganglia of patients with a *PINK1* mutation have reported an increase in myo-inositol by using MR spectroscopy [35]. Myo-inositol is purported to be a marker of glial cell death and neuroinflammation [36]. It is noteworthy that monoglycerides and myo-inositol are also downstream products of the IP_3_-DAG signalling pathway [37]. This pathway plays a role in facilitating release of Ca^2+^, which is important in cellular growth and synaptic plasticity [37].

Our results are in line with some of the findings from other studies. Lu et al. studied the changes in metabolites in the brains of goldfish treated with MPTP. ^1^H NMR-based metabolomics revealed an increase of myo-inositol and linoleic acid in the PD brain [17]. In another independent study, a paraquat-treated *Drosophila* model was used to elucidate changes of metabolites. It was shown that myo-inositol, 1-monopalmitin, 1-monostearin and the fatty acids palmitate and oleate were increased in the heads of the paraquat-treated flies [38]. In these studies, however, specific changes in the midbrain only were not determined. Furthermore, it must be noted that the findings from this study must be further validated for specificity to dopaminergic loss. This can be confirmed using a lesion that spares dopaminergic neurons but selectively damages surrounding neurons, such as an excitotoxic lesion of the striatum.

## 4. Materials and Methods

Twenty-six, four to six weeks old male Sprague-Dawley rats were purchased from the Laboratory Animal Unit at The University of Hong Kong. All experimental procedures were in accordance with the Committee on the Use of Live Animals in Teaching and Research of The University of Hong Kong (3491-14). The animals weighed 200 g at the beginning of treatment and were housed in pairs, in a temperature-controlled room with a 12-h dark/light cycle and free access to food and water.

All chemicals and reagents were obtained from Sigma Aldrich (United Kingdom), unless stated otherwise.

### 4.1. Stereotactic Injection of 6-OHDA

The rats were randomly divided into sham (n = 13) and 6-OHDA (n = 13) groups. Fresh stock solution (3 μg/μL) of 6-hydroxydopamine hydrobromide was prepared in saline (0.9% *w*/*v* NaCl) containing 0.2 mg/mL ascorbic acid. Rats were anaesthetized with 60 mg/kg pentobarbital (Alfansan International, Netherlands). 12 μg (in 4 μL) of 6-OHDA or vehicle was introduced into the right medial forebrain bundle (MFB) of the rat, using a Hamilton syringe connected to a 33G needle, at the rate of 1 μL/min. The coordinates of the injection site were: ML = −1.2, AP = −4 and DV = +7.5 (below dura), with the nose bar position at 4.5, based on the atlas by Paxinos and Watson. These coordinates were slightly modified from the study by Torres et al. [39]. Sham rats were injected with the same volume (4 μL) of vehicle. The needle was left in place for five minutes before retracting, and the incision was sutured. Body temperature and heart rate of the animals was measured constantly throughout the procedure.

### 4.2. Behavioural Assessment 

At two weeks post-surgery, behaviour assessment for motor function was carried out in an isolated room, in the following order:

***Cylinder test:*** The protocol used by Schallert et al. [40] was modified slightly. Rats were placed in a transparent acrylic cylinder for a total of three minutes and recorded. During every rear, the use of ipsilateral, contralateral or both forelimbs was counted, for a minimum of three and a maximum of ten rears or three minutes, whichever was first. The cylinder was cleaned with 70% ethanol between each use. Results were expressed as % trials with ipsilateral use only.

***Apomorphine-induced rotation test***: Rats were injected subcutaneously with 0.3 mg/kg of apomorphine hydrochloride dissolved in saline. Five minutes after injection, each rat was placed in a cylinder and recorded for 40 min. The number of contralateral rotations in 30 min was measured. Minimum four rotations per minute was considered as acceptable criteria for a successful model. Only the rats that were successfully lesioned based on this criterion were used for further studies.

### 4.3. Immunohistochemistry 

After behavioural assessment, the mesencephalon of five rats from each group were harvested for immunohistochemical staining of tyrosine hydroxylase as follows:

***Tissue processing and frozen-sectioning:*** Rats were overdosed with 150–200 mg/kg pentobarbital, followed by intra-cardiac transfusion of ice-cold saline and subsequently, freshly prepared ice-cold 4% paraformaldehyde (PFA) in 0.1 M phosphate-buffer. The substantia nigra *pars compacta* (SNpc) was then dissected out, post-fixed and soaked in increasing concentrations of sucrose. The tissue was then snap-frozen and stored in −80 °C until use. Thin slices of 15 μM were the sectioned using on a cryostat (Leica, Germany) and mounted. Every 6th section of the mesencephalon was collected, dried and stored at 4 °C until use.

***DAB staining and imaging:*** Sectioned tissues were washed with 0.1 M PBS thrice, followed by blocking of endogenous peroxidase activity with 30% hydrogen peroxide in methanol for 30 min. Tissues were then incubated with the anti-tyrosine hydroxylase biotin-conjugated antibody (1:400, Cell Signaling Technologies, Danvers, MA, USA), in a humid slide chamber at 4 °C overnight. Following anti-biotin secondary antibody (1:400, Dako, USA) incubation, slides were stained using 3,3′-diaminobenzidine (DAB) solution from the ABC staining kit (Invitrogen, USA) according to the manufacturer’s protocol. Brain sections were then counter stained with hematoxylin, dehydrated with ethanol and toluene and mounted. Slides were observed at a magnification of 5× using Brightfield microscopy (Zeiss Axioplasm, Germany), and stitched using the Image Composite Editor software (Microsoft, Albuquerque, NM, USA). Cell counting was then done using ImageJ (National Institute of Health, Bethesda, MD, USA). Cell counts were expressed as a % of the right side to the left side.

### 4.4. Tissue Harvest 

After behavioural assessment, rats were asphyxiated using CO_2_ and tissues were harvested for metabolomics analysis as follows:

***Plasma extraction:*** the plasma was extracted form a total of 10 rats for each group. Briefly, one millilitre of blood was drawn by intra-cardiac transfusion, using an EDTA-buffer coated syringe. The needle was taken off and blood transferred to Eppendorf tubes and shaken. Samples were kept on ice, and then centrifuged at 4500 rpm in a 4 °C Eppendorf centrifuge for 15 min. The supernatants were collected, and samples were stored at −80 °C until use.

***Microdissection of brain:*** The brain of eight rats from each group were harvested and briefly rinsed in ice cold 0.1 M phosphate-buffered saline (PBS) to remove any excess blood. It was then slit down the middle to divide the right and left sides and the cerebellum and entire mesencephalon tissues were separated according to our previous protocol [41], snap frozen in liquid N_2_ and stored in −80 °C until use.

***Liver:*** A part of the liver from eight rats in each group was cut and briefly rinsed in ice cold 0.1 M PBS to remove any excess blood. It was then snap frozen in liquid N_2_ and stored in −80 °C until use.

### 4.5. Sample Extraction for Metabolomics

***Plasma sample extraction:*** In vial dual extraction (IVDE) was slightly modified based on our previous protocol [42]. Briefly, 20 μL of LC-MS grade water was added to 40 μL plasma, followed by 80 μL of LC-MS grade methanol containing 10 μg/mL of succinic- d4- acid as internal standard (IS). After vortex, 400 μL of LC-MS grade methyl tertiary butyl ether (MTBE) with 10 μg/mL of tripentadecanoin as internal standard was added and then the samples were mixed thoroughly. Following a final addition of 100 μL LC-MS grade water, samples were centrifuged at 3000× *g* for 10 min at 4 °C to give a clear separation of MTBE (upper) and aqueous (lower) phases with protein aggregated at the bottom. The aqueous and MTBE layers were collected and stored until analysis at −20 °C and −80 °C, respectively.

***Brain and liver sample extraction:*** IVDE was slightly modified based on our previous protocol [43]. Prior to homogenisation, 5 μL of methanol and 5 μL of IS (50 μg/mL succinic-d4 acid in 80% methanol) was added per milligram of tissue. The tissue was then homogenised using a Tissuelyzer (Qiagen, Germany) for ten cycles of 30 s at 25 Hz. Subsequently, 80 μL of homogenate was diluted with 120 μL of methanol. The subsequent extraction procedure was similar to plasma extraction, with addition of 40 μL of water, 1000 μL of MTBE containing tripentadecanoin (10 μg/mL) and thorough vortex. After addition of 160 μL of water, samples were then centrifuged at 3000× *g* for 10 min at 4 °C. The aqueous and MTBE layers were then separated and stored.

### 4.6. Derivatization of Tissues for GC-MS Analysis

Roughly 20 μL of plasma/brain/liver sample was dried under a stream of N_2._ For plasma, 50 μL of O-methoxyamine-HCL (MOX) in pyridine (20 mg/mL) was added to the residue and maintained at 70 °C for 30 min. Samples were then dried again and reconstituted in a 1:1 (*v*/*v*) solution of acetonitrile and the derivatizing agent BSTFA (1% TMCS). The derivatization process was operated at 70 °C for an hour. For brain and liver tissues, the residues were reconstituted in a 1:1 (*v*/*v*) solution of acetonitrile and the derivatizing agent BSTFA (1% TMCS) directly and incubated at 37 °C for an hour. All the resulting derivatized samples were transferred to amber HPLC vials with inserts for GC-MS analysis.

### 4.7. GC-MS Analysis

GC-MS analysis was carried out on a Shimadzu GC-2010 Plus gas chromatograph equipped with a GCMS-QP2010 SE single quadruple mass spectrometer (Shimadzu, Japan). Sample (0.5 μL) was injected on a BP5MS (5% phenyl polysilphenylene-siloxane) capillary column (length 30 m, thickness 0.25 mm, diameter 0.25 mm) in the split mode with a split ratio of 1:60. The gradient temperature started from 60 °C and was held for 1 min, followed by a linear increase of 10 °C/min to 320 °C. Then, it was kept at 320 °C for 4 min. The carrier gas (helium) flow rate was set at 40 cm/s. Mass spectra analysis was performed using electron impact ionisation of 70 eV with an ion-source temperature of 200 °C, an interface temperature of 320 °C and an injection temperature of 280 °C. Data were collected between *m*/*z* 50–600 Da in a SCAN mode. Quality control (QC) samples made from pooled corresponding samples were injected periodically.

### 4.8. Data Processing and Metabolite Identification

The raw data generated was converted to mzXML using the GC-MS Postrun Analysis software (Shimadzu, Japan). The mzXML files were further processed for peak picking (signal to noise threshold = 5) and retention time correction using the XCMS package in R. The pre-processed metabolomics data were normalized separately in R which resulted in the best clustering of QC samples in principal component analysis (PCA) score plot. All semi-quantification was done using raw peak areas of selected features normalized to that of internal standard. Metabolite identification was done by comparing the GC-MS fragmentation mass spectra to those found in the National Institute of Standards and Technology (NIST) database. Identification of metabolites was confirmed by comparing the retention time and mass spectrum to pure standards if the similarity index was less than 90%.

### 4.9. Statistical Analysis

SIMCA (Umetrics, Sweden) was used for multivariate statistical analysis. Principle component analysis (PCA) was performed on the plasma and midbrain samples combined to assess reproducibility of the data. Orthogonal partial least squares-discriminant analysis (OPLS-DA) plots were then built for both the plasma and mesencephalon tissues after Pareto scaling and excluding features with VIP values <1. Corresponding S-plots were used for feature selection. Since the lesion for mesencephalon tissues was unilateral, the metabolite levels on the lesioned side were normalized to the intact side and these ratios were subsequently used for OPLS-DA plots.

Following feature selection, univariate analysis was performed on semi-quantified data using GraphPad Prism (GraphPad, USA). Mann-Whitney test followed by Benjamini and Hochberg correction (*q* value < 0.05) were used to find statistically significant features. Significant midbrain features were also measured in the cerebellum. Spearman’s correlation test, followed by Benjamini and Hochberg correction was performed to assess correlation with the behaviour assessment. Post-hoc power analysis was performed in R with package “pwr”. Heat map cluster analysis was performed in R with package “ggplot”. All data is expressed at mean ± S.D.

## 5. Conclusions

In summary, this study used metabolomics to elucidate changes in 6-OHDA-induced parkinsonism. Two saturated free fatty acids, palmitic and stearic acid, were increased in the plasma of rats that underwent 6-OHDA injection. Monopalmitin, monostearin and myo-inositol showed an asymmetric distribution between the ipsilateral and contralateral mesencephalon. Changes of the midbrain metabolites may be associated with neuronal loss elicited by 6-OHDA while palmitic acid and stearic acid showed a high correlation with behaviour tests, indicating a possible association with disease severity.

## Figures and Tables

**Table 1:** Summary of correlation results of plasma and midbrain metabolites with the cylinder test.

Metabolite	Site	Spearman’s Correlation Coefficient	*q*-Value
Palmitic acid	Plasma	0.674	**0.035**
Stearic acid	Plasma	0.649	**0.027**
Monopalmitin	Midbrain	−0.578	0.07
Monostearin	Midbrain	−0.439	0.205
Myo-inositol	Midbrain	−0.205	0.438

## Supplementary Materials

The following are available online at http://www.mdpi.com/2218-1989/9/2/31/s1.
