# Supplementary material for: Palmitate and Stearate are Increased in the Plasma in a 6-OHDA Model of Parkinson’s Disease
Source: Metabolites. 2019 Feb 13;9(2):31. doi: 10.3390/metabo9020031 (PMC6409985; doi:10.3390/metabo9020031)
Supplement: Supplementary file 1 [file metabolites-09-00031-s001.pdf]

Supplementary material

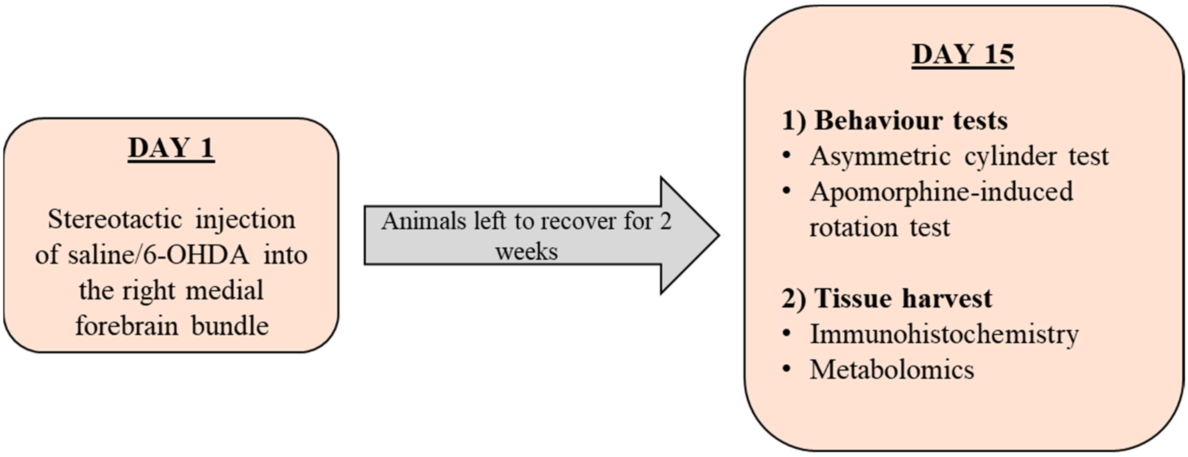

Supplementary figure 1. Diagrammatic representation of the study design.

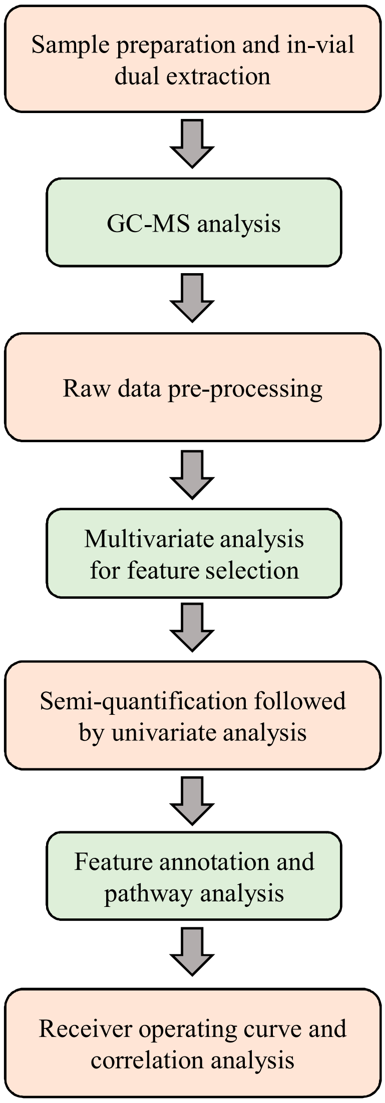

Supplementary figure 2. Schematic of the metabolomics study design. Workflow indicates the experimental design starting from sample preparation for GC-MS analysis, feature selection using multivariate and univariate analysis, and finally feature identification.

A.

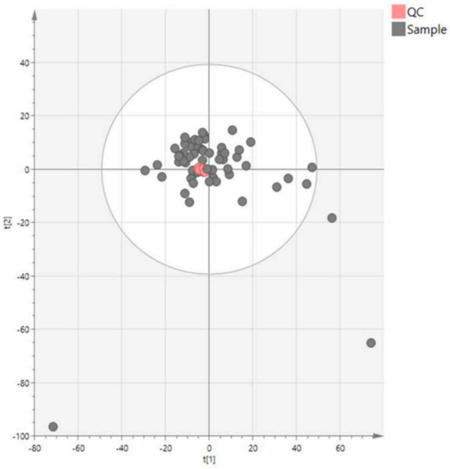

B.

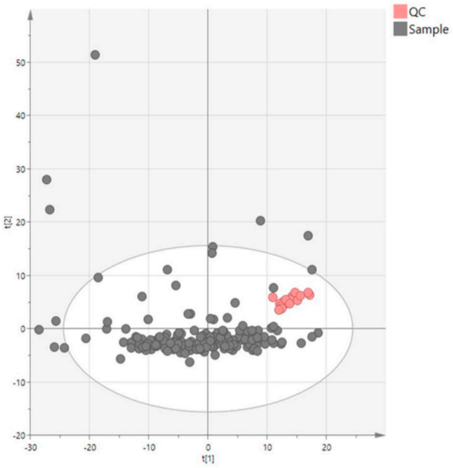

Supplementary figure 3. PCA plots showing clustering of QC samples. PCA plots for plasma (A) and mid-brain (B), illustrating a clear clustering of the QC samples.

A.

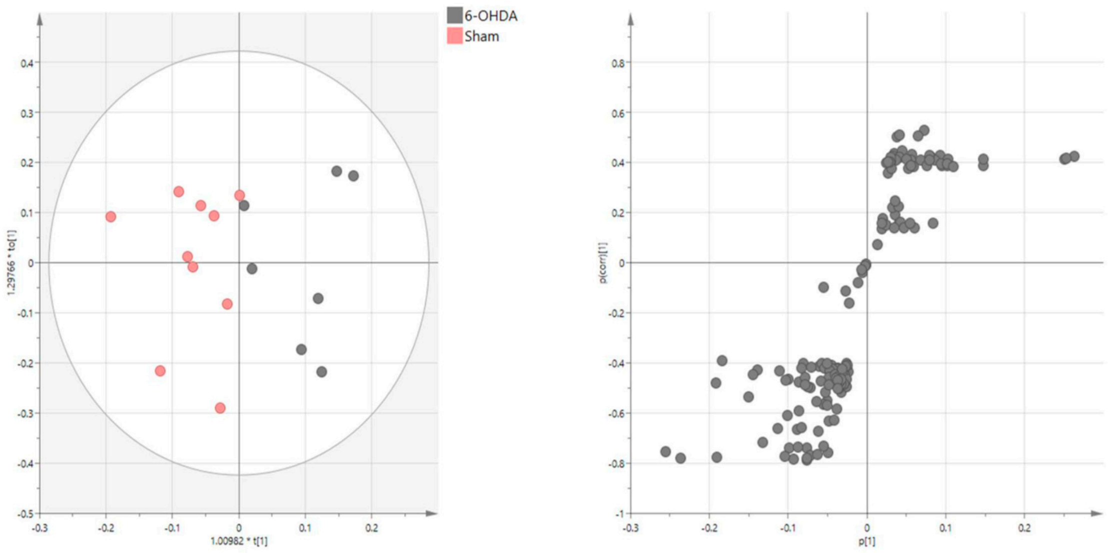

B.

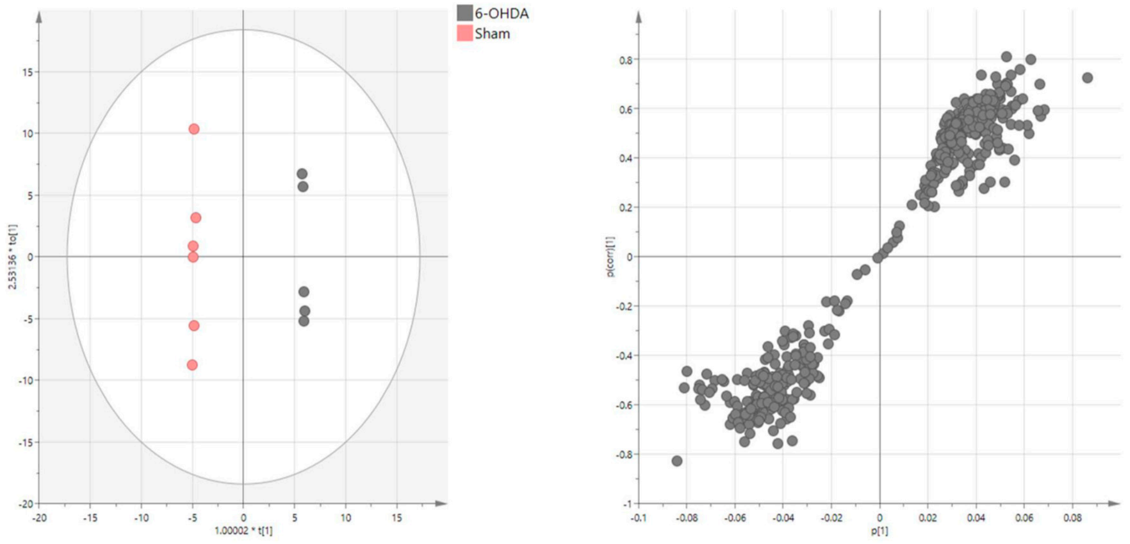

Supplementary figure 4. OPLS-DA score plot and S-plot for plasma and mid-brain samples. OPLS-DA plots (left) showing a separation between the Sham and 6-OHDA groups in the plasma (A) and mid-brain (B), along with their corresponding S-plots (right) indicating thresholds for features selected.

A.

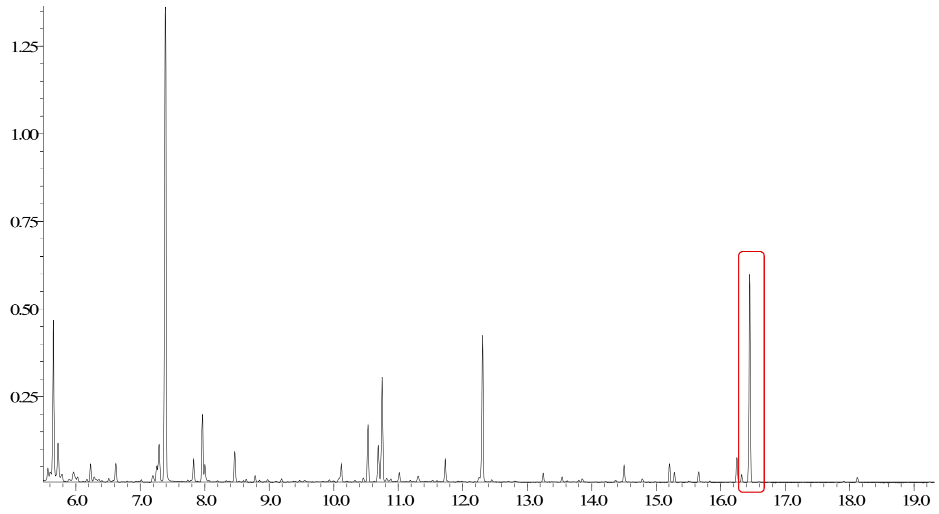
1.25
1.00
0.75
0.50
0.25
6.0 7.0 8.0 9.0 10.0 11.0 12.0 13.0 14.0 15.0 16.0 17.0 18.0 19.0

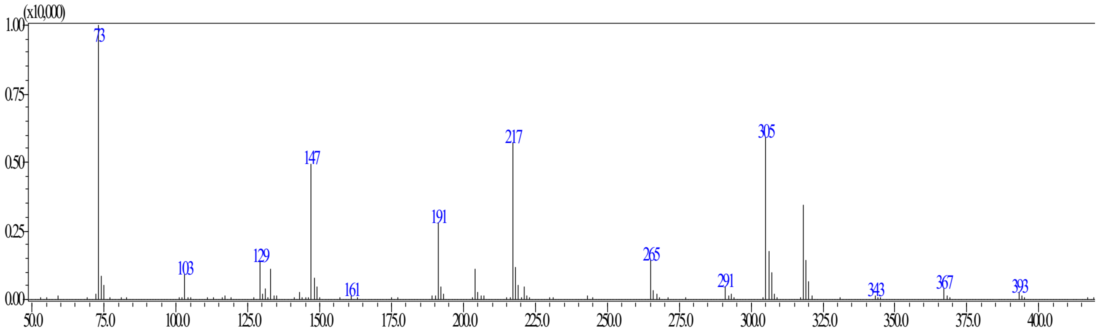
(x10,000)
1.00
0.75
0.50
0.25
0.00
73
103
129
147
161
191
217
265
291
305
343
367
393
50.0 75.0 100.0 125.0 150.0 175.0 200.0 225.0 250.0 275.0 300.0 325.0 350.0 375.0 400.0

B.

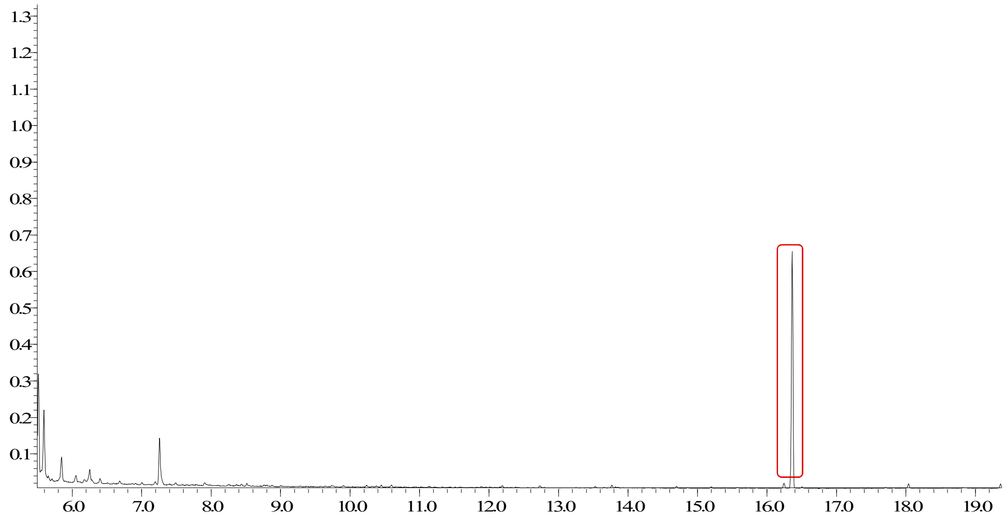

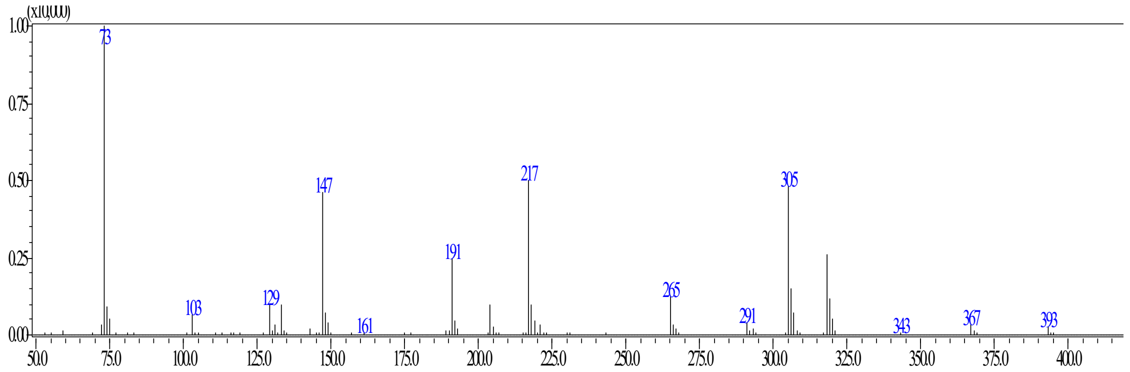

Supplementary figure 5. Chromatograms and mass spectra of myo-inositol. Highlighted myo-inositol peak (top figure) and its corresponding mass spectrum (bottom figure) from a midbrain sample (A) and the myo-inositol reference standard (B).

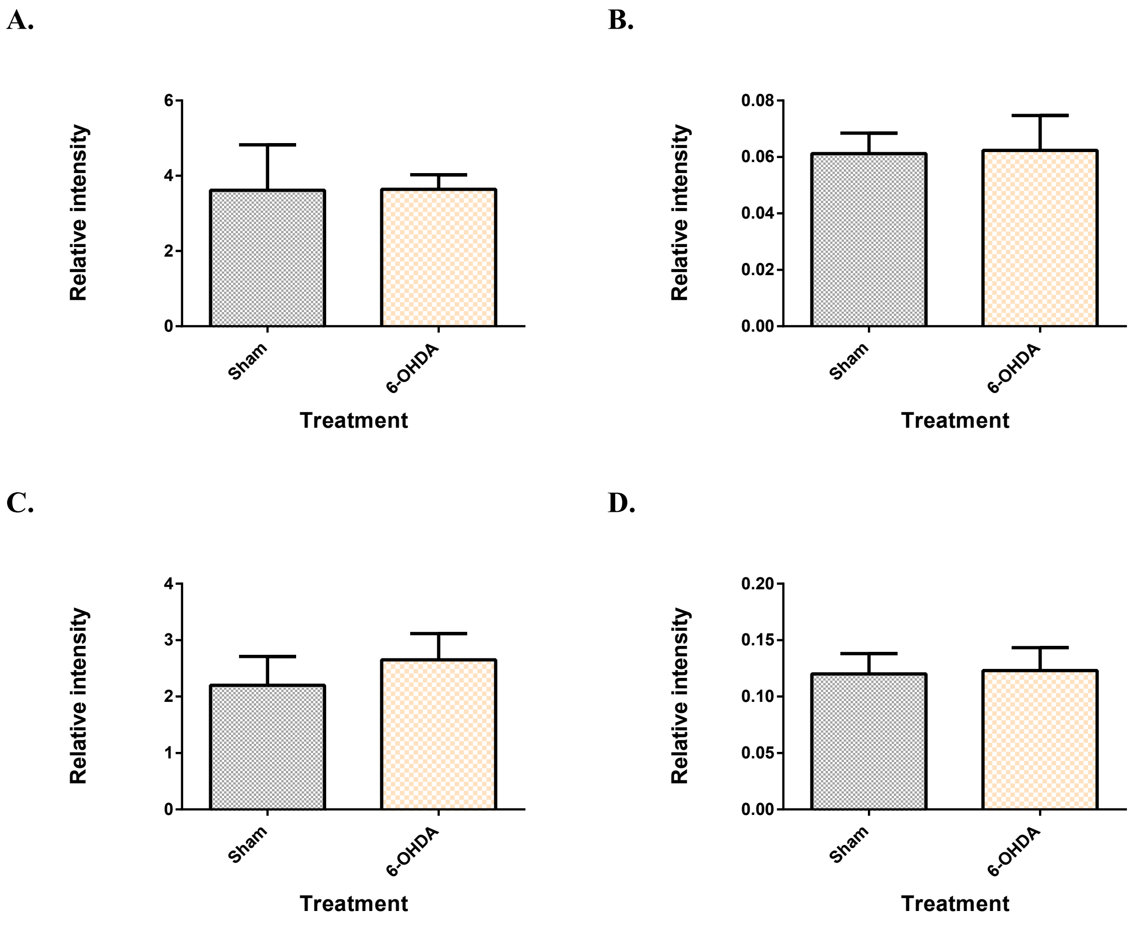

Supplementary figure 6. Changes in liver metabolites. Palmitic acid (A), monopalmitin (B), stearic acid (C) and monostearin (D) were all unchanged in the livers of the 6-OHDA vs sham groups. Data represent mean ± S.D of at least 5 animals in each group.
